# Perfectionistic Discrepancy, Stress, and Preference for Ugly–Cute Memes: Evidence from a Survey and a Randomized Experiment

**DOI:** 10.3390/bs16091570

**Published:** 2026-09-04

**Authors:** Yi An, Yu Jin, Ji Xu, Tiansheng Xia, Dingbang Luh

**Affiliations:** 1 School of Art and Design, Guangdong University of Technology, Guangzhou 510006, China; 2 Design Innovation Service Department, Industrial Culture Development Center, Ministry of Industry and Information Technology, Beijing 100846, China

**Keywords:** perfectionistic discrepancy, ugly–cute memes, stress, state anxiety, emotional compensation

## Abstract

Ugly–cute memes, which blend visual incongruity with cuteness, have become a prominent form of nonconventional online esthetics on social media, yet little is known about who prefers them and why. The present research examined how perfectionistic discrepancy and stress-related states relate to ugly–cute meme preference across two studies. In Study 1, 356 Chinese adults completed the Discrepancy subscale of the Almost Perfect Scale–Revised, together with measures of perceived stress, state anxiety, and preference for a pretested ugly–cute meme. The mediation model yielded a negative direct association between perfectionistic discrepancy and ugly–cute meme preference, as well as positive indirect associations through state anxiety and through the serial path from perceived stress to state anxiety. Study 2 used a randomized recall-based stress induction with 393 valid participants. Compared with participants in the neutral-recall control condition, those in the stress-recall condition reported higher state anxiety, rated ugly–cute images more favorably, and were more likely to choose an ugly–cute image in the forced-choice task. The estimated indirect effect of the stress manipulation on ugly–cute image preference through state anxiety was statistically significant. Overall, stress-related states appear to increase the appeal of ugly–cute content. Perfectionistic discrepancy, in turn, relates to this preference through opposing direct and indirect routes.

## 1. Introduction

Internet memes are cultural units formed through the dissemination, imitation, and recreation of content by Internet users (Shifman, 2013). At the level of visual culture, online esthetics do not always follow conventional ideals of refinement and harmony, nor do they favor easily processed forms. Rough, anti-normative, and even ugly visual forms may also develop into stable esthetic categories and acquire communicative appeal in online environments (Douglas, 2014; Schonig, 2020). Against this background, ugly–cute memes, which combine incongruity with cuteness, have appeared widely in sticker packs, online character images, cultural and creative products, and designer toys. They spread rapidly among young people, providing an important context for observing contemporary nonstandardized esthetic preferences (Li et al., 2024). For example, Labubu, an ugly–cute character marketed through blind-box products by the Chinese company Pop Mart, has recently attracted considerable attention in global collector markets (Pu, 2025), illustrating the commercial reach of ugly–cute esthetics. Research on ugly–cute dolls also suggests that their appeal to Generation Z consumers may be partly driven by needs for individuality and differentiation from others (C. Zhao, 2026). However, existing research has mainly focused on the visual features of ugly–cute stimuli and the emotional responses they evoke, leaving open the question of who is drawn to such content and by what psychological route.

Ugly–cute memes are online, image-based cultural units that combine, within a single figure, cuteness cues and perceptual incongruity. They retain baby-schema features (such as a large head, large eyes, and rounded contours) that elicit caretaking tendencies (Glocker et al., 2009; Marcus et al., 2017). At the same time, they depart conspicuously from conventional standards of proportion, symmetry, or expressive coherence, which lowers processing fluency (Reber et al., 2004; Veryzer & Hutchinson, 1998). Together, these two properties make ugly–cute memes approachable and low in threat. Yet they remain novel enough to require reappraisal. Conventional cute content carries cuteness cues without incongruity and is processed fluently. It therefore elicits a uniformly positive response. The evaluation of ugly–cute memes, by contrast, is internally conflicted. Here an initial ugliness judgment must be reappraised in terms of humor, interest, or distinctiveness (Graf & Landwehr, 2017; Leder et al., 2004).

Perfectionistic discrepancy provides an important perspective for understanding individual differences in the appeal of ugly–cute memes. Perfectionism is a multidimensional disposition that is usually resolved into two superordinate dimensions: perfectionistic strivings and perfectionistic concerns (Stoeber & Otto, 2006). Perfectionistic discrepancy is the facet that carries the concerns dimension: the perception that one consistently fails to meet the standards one has set, together with the dissatisfaction and self-critical evaluation that follow (Slaney et al., 2001, 2002). Because ugly–cute memes visibly depart from conventional standards of proportion, symmetry, and expressive coherence, this facet gives reason to expect that individuals higher in perfectionistic discrepancy will like them less. However, ugly–cute objects are not merely ugly; they combine the ugliness arising from visual incongruity with retained cuteness (Marcus et al., 2017).

The esthetic pleasure–interest model proposes that esthetic liking may arise not only from the pleasure associated with processing fluency but also from interest triggered by novelty, complexity, and meaning exploration (Graf & Landwehr, 2017). Recent research has found that the appeal of ugly–cute memes is driven primarily by the pleasure elicited by humor, whereas perceived ugliness alone does not elicit a comparable positive response (Li et al., 2024). This finding is also compatible with accounts that treat ugliness as a distinct and complex esthetic experience (Felisberti, 2022) and cuteness as an ambivalent esthetic category (Ngai, 2012). Thus, two opposite associations may coexist between perfectionistic discrepancy and ugly–cute meme preference. Ugly–cute memes are a particularly suitable case for observing these opposing associations because they combine two properties that most other unconventional esthetic categories do not hold together. Their departure from standards of refinement and symmetry engages individuals who are sensitive to discrepancy on the evaluative side, which supports a negative direct path. At the same time, their retained cuteness keeps them low in threat and approachable. Cuteness cues of this kind reliably elicit caretaking and affiliative tendencies (Glocker et al., 2009). The cuteness response has also been characterized as positive, unthreatened, and approach-motivated (Nittono, 2016). Such content is therefore available for emotional repair, which supports a positive indirect path. By contrast, a category such as grotesque imagery deviates from the same standards through aversive perceptual qualities that are appraised as disgusting. Disgust is an avoidance-oriented esthetic emotion that motivates withdrawal from its object (Silvia & Brown, 2007). Cuteness and disgust have indeed been argued to shift social-engagement motives in opposite directions, with cute entities drawing approach and disgusting ones prompting disengagement (Sherman & Haidt, 2011). Grotesque imagery would therefore not be expected to sustain the compensatory route in the same way.

The compensatory side of this account rests on a further premise. Individuals with high perfectionistic discrepancy are more likely to experience elevated stress and anxiety (Gnilka et al., 2012; Simon et al., 2025), which may increase the appeal of content that offers emotional relief. Humorous memes may support coping with negative experiences (Akram & Drabble, 2022), and the compensatory consumer behavior model suggests that self-discrepancy and aversive psychological states can motivate individuals to seek objects with symbolic or emotional value (Mandel et al., 2017). Because ugly–cute memes combine humor and cuteness while remaining relatively low in threat, they may be especially appealing under heightened stress and anxiety. Together, these considerations suggest that perfectionistic discrepancy may be positively associated with ugly–cute meme preference indirectly through perceived stress and state anxiety.

To test this possibility, we conducted two complementary studies. Study 1 was a cross-sectional survey of 356 Chinese adults that tested a serial mediation model in which perfectionistic discrepancy relates to ugly–cute meme preference both directly and indirectly through perceived stress and state anxiety. Study 2 was a randomized between-subjects experiment with 393 participants in which stress was manipulated through an autobiographical recall task and preference was assessed with both continuous ratings and forced choices.

## 2. Study 1

### 2.1. Hypotheses

#### 2.1.1. Perfectionistic Discrepancy and Ugly–Cute Meme Preference

The Almost Perfect Scale–Revised operationalizes multidimensional perfectionism through three subscales: High Standards, Order, and Discrepancy (Slaney et al., 2001). High Standards indexes perfectionistic strivings, and Order indexes a preference for neatness, whereas Discrepancy carries the perfectionistic-concerns dimension and is routinely treated as the defining marker of maladaptive perfectionism (Rice & Ashby, 2007). Psychometric evidence from two independent samples supports assessing this construct with the original Discrepancy subscale, which retains discriminant validity and shows stable associations with criterion variables including stress and emotion regulation (Rice et al., 2019). The subscale thus indexes a unified evaluative and affective orientation toward what falls short of an internalized standard, an orientation that applies to the appraisal of objects as well as of the self.

Perfectionistic discrepancy describes a habitual comparison between an actual state and an internalized standard, in which attention is drawn to the shortfall and the shortfall is experienced as unsatisfactory. This evaluative orientation is not confined to appraisals of the self. In consumer research, it is the discrepancy facet rather than the standards facet that drives responses in situations where a perceived gap between an ideal and an actual state is at stake (Chang et al., 2021). Conventional esthetic evaluation, in turn, rewards symmetry, proportion, and typicality, all of which raise processing fluency and, with it, liking (Reber et al., 2004; Veryzer & Hutchinson, 1998). Ugly–cute memes are constructed from precisely the deviations that such an orientation registers as shortfalls, namely exaggerated proportions, asymmetrical features, and incongruous expressions. Consistent with this reasoning, consumers higher in perfectionism are less willing to accept products with flawed appearance (Chen et al., 2023), and perfectionism dimensions predict heightened concern with appearance symmetry (Toh et al., 2022). Individuals higher in perfectionistic discrepancy should therefore evaluate ugly–cute memes less favorably on the evaluative route.

**H1.**  *Perfectionistic discrepancy is negatively associated with preference for ugly–cute memes after controlling for perceived stress and state anxiety.*

#### 2.1.2. The Mediating Role of Perceived Stress

Perceived stress refers to an individual’s subjective evaluation of whether environmental demands exceed coping resources, emphasizing uncontrollability, unpredictability, and overload in life events (Cohen et al., 1983; Lu et al., 2017). The association between perfectionistic discrepancy and ugly–cute meme preference may arise not only from esthetic evaluation but also indirectly through perceived stress. Individuals high in perfectionistic discrepancy continuously attend to the gap between their internalized standards and their actual state, and this monitoring is itself a source of appraised demand. Studies using the Almost Perfect Scale–Revised show that individuals classified as maladaptive perfectionists on the basis of Discrepancy report higher perceived stress than adaptive perfectionists (Ashby et al., 2012), and that Discrepancy positively predicts stress while High Standards does not predict it in the same way (Simon et al., 2025). In a multi-site sample of 705 Chinese undergraduates, perfectionistic discrepancy was likewise associated with lower perceived coping and more negative stress appraisal (Xu et al., 2026). At the level of the superordinate perfectionistic-concerns dimension, which Discrepancy indexes, self-critical perfectionism has been linked to heightened daily stress reactivity and to psychopathology more broadly (Dunkley et al., 2014; Limburg et al., 2017). Consistent with the compensatory consumer behavior model introduced above (Mandel et al., 2017), as perfectionistic discrepancy is associated with greater perceived stress, individuals may become more receptive to low-threat ugly–cute content with humorous or emotionally soothing qualities.

**H2.**  *Perceived stress mediates the relationship between perfectionistic discrepancy and preference for ugly–cute memes.*

#### 2.1.3. The Mediating Role of State Anxiety

State anxiety refers to the tension, worry, and uneasiness experienced at a given moment. It fluctuates with situational demands (Spielberger et al., 1983). Perfectionistic discrepancy is the facet most consistently associated with anxiety. Individuals classified as maladaptive perfectionists on the basis of Discrepancy report the highest anxiety levels among perfectionist subtypes (Gnilka et al., 2012). Discrepancy positively predicts anxiety, whereas High Standards shows no significant effect on it (Simon et al., 2025), and in a laboratory-task study of 83 high-achieving university students, higher discrepancy was associated with greater task-related and overall anxiety (Rassaby et al., 2021). Appearance-related research at the level of the broader construct further suggests that anxiety and depression may mediate the associations of perfectionism with self-perceptions of orofacial appearance (Gao et al., 2023). Studies of mental-health and crisis-related memes suggest that humorous memes may help individuals make sense of negative experiences and alleviate emotional distress (Akram & Drabble, 2022; Cancelas-Ouviña, 2021), while cute elements may also contribute to positive emotional responses (Chou et al., 2022). According to mood management theory, individuals experiencing negative affective states tend to select media content with the potential to improve their current mood (Zillmann, 1988). Experimental evidence indicates that viewing memes elicits stronger cuteness and humor responses, and more positive emotion. This emotional response in turn predicts greater coping efficacy (Myrick et al., 2022). Therefore, when individuals have higher state anxiety, they may place greater value on the humor, cuteness, and low-threat qualities of ugly–cute memes, forming a positive indirect association between perfectionistic discrepancy and ugly–cute meme preference through state anxiety.

**H3.**  *State anxiety mediates the relationship between perfectionistic discrepancy and preference for ugly–cute memes.*

#### 2.1.4. The Serial Mediating Role of Perceived Stress and State Anxiety

**Figure 1:**
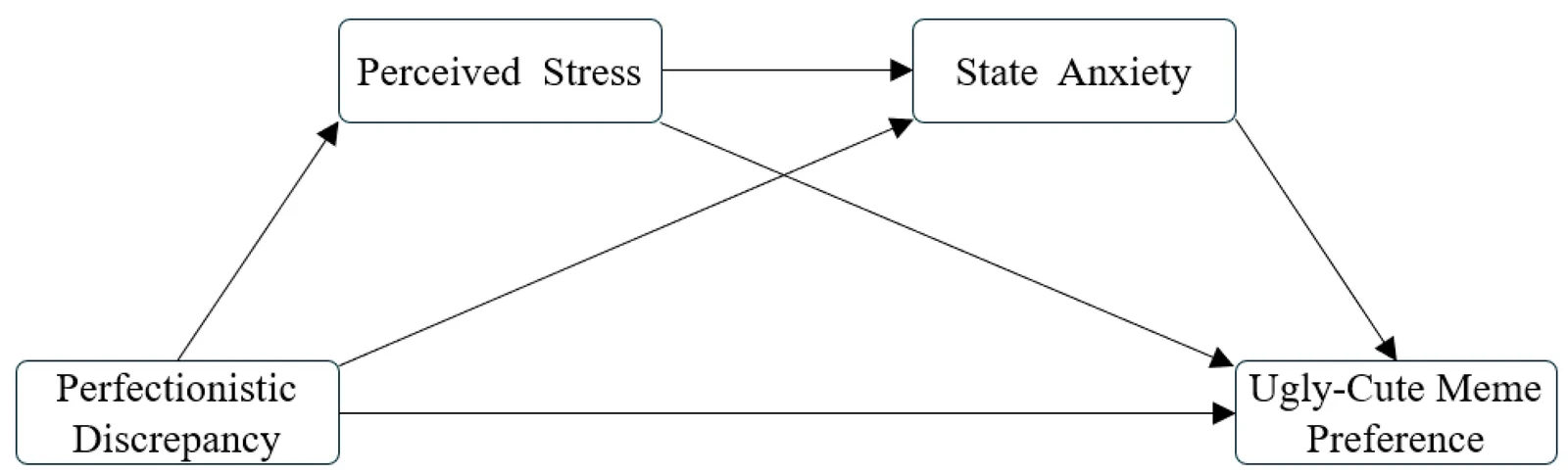
The theoretical model.

Perceived stress and state anxiety are closely related. Higher stress appraisals are typically accompanied by stronger feelings of tension, uneasiness, and worry (Liu et al., 2021; Lu et al., 2017). Perceived stress mainly reflects individuals’ cognitive evaluation of whether external demands and coping resources are imbalanced, whereas state anxiety is a more proximal emotional response that may be triggered by such threat appraisal (Cohen et al., 1983; Spielberger et al., 1983). Longitudinal research also shows that perfectionism moderates the effect of chronic stress on depression (Békés et al., 2015), suggesting a stable link between perfectionistic concerns and the stress–negative emotion chain in which discrepancy is located. Xiong et al. (2024) similarly reported that adaptation difficulties among students with high maladaptive perfectionism, across academic and interpersonal domains, may induce anxiety and other psychological distress. Perceived stress is thus positioned upstream of state anxiety in this emotional chain. If stress is further processed into negative emotional states, it may be more likely to translate into a preference for ugly–cute memes. As shown in Figure 1, perfectionistic discrepancy is modeled as relating to ugly–cute meme preference through one negative direct path and three positive indirect paths: through perceived stress, through state anxiety, and through the serial chain from perceived stress to state anxiety.

**H4.**  *Perceived stress and state anxiety serially mediate the relationship between perfectionistic discrepancy and preference for ugly–cute memes.*

### 2.2. Participants and Design

Using convenience sampling, questionnaires were distributed via the Wenjuanxing online survey platform to 380 Chinese adults who had used ugly–cute memes online. The questionnaire included demographic information, an image evaluation task, and scales measuring perfectionistic discrepancy, perceived stress, and state anxiety. Fifteen responses were removed because participants failed attention checks, six because of abnormal completion times, and three because of missing key information. A final sample of 356 valid responses was obtained, yielding a valid response rate of 93.68%. Among the valid participants, 190 were women (53.37%) and 166 were men (46.63%). The mean age was 21.72 years (*SD* = 2.14), and the sample mainly consisted of college students. All participants read the study information and provided informed consent before completing the survey. The research procedure followed the principles of anonymity, voluntariness, and confidentiality and was approved by the Departmental Ethics Committee and the Institutional Review Board of the Guangdong University of Technology (approval number: GDUTXS20260207). Demographic information is shown in Table 1.

We adopted a Monte Carlo power analysis designed for indirect effects (Schoemann et al., 2017) to determine the minimum required sample size. Small to moderate correlations between variables were assumed in the model. The specified parameters were statistical power = 0.80, confidence level = 99%, 10,000 Monte Carlo replications, and 20,000 bootstrap draws per replication. The power analysis indicated that the minimum required sample size for this setting was 287. The final sample therefore exceeded this requirement.

### 2.3. Stimuli

The stimuli in Study 1 were existing memes that were circulating on Chinese social media platforms. Six candidate images were collected, including three with typical esthetic styles and three with ugly–cute styles. Before formal evaluation, the candidate images were standardized in size and resolution, simplified in background, and matched for brightness and color saturation to reduce the influence of irrelevant physical attributes. A separate sample of 30 participants, drawn from the same population as the main study, rated the six candidate images. Participants rated each image on beauty, degree of ugly–cuteness, and preference using the items “This image is beautiful,” “This image is ugly–cute,” and “How much do you like this type of image,” respectively, all on five-point Likert scales. To guard against confounding by extraneous attributes, the candidate images were also screened for familiarity and visual complexity, and the two images finally selected were comparable on these attributes. Based on the combined ratings, one image with high beauty and low ugly–cuteness was selected as the control stimulus, and one image with low beauty and high ugly–cuteness was selected as the target ugly–cute stimulus. Paired-samples *t*-tests showed that the control stimulus was rated as significantly more beautiful than the ugly–cute stimulus, *t*(29) = 4.94, *p* < 0.001, and significantly lower in ugly–cuteness, *t*(29) = −7.75, *p* < 0.001. The two selected images were then used in the formal evaluation task, and participants’ preference ratings for the target ugly–cute stimulus served as the dependent variable.

### 2.4. Procedure and Measures

Established scales were used to measure perfectionistic discrepancy, perceived stress, and state anxiety, and a single item was used to measure preference for ugly–cute memes. The sources, number of items, sample items, and internal consistency of each measure are shown in Table 2. For all multi-item variables, item means were used as scores. Reverse-scored items were recoded before analysis. Higher scores indicated higher levels of the corresponding variable. Discrepancy was rated on a seven-point scale (1 = strongly disagree, 7 = strongly agree), the perceived stress items on a five-point scale (1 = never, 5 = very often), the state anxiety items on a four-point scale (1 = not at all, 4 = very much so), and the single preference item on a five-point scale (1 = not at all, 5 = very much so).

Perfectionistic discrepancy was measured using the Discrepancy subscale of the Almost Perfect Scale–Revised (APS-R; Slaney et al., 2001), which reflects individuals’ perceived discrepancy between actual performance and internal standards, as well as dissatisfaction, frustration, and self-criticism resulting from this discrepancy. This subscale was selected because the discrepancy facet indexes the perfectionistic-concerns dimension and is the component that directly concerns the perceived deviation of an object or outcome from an internalized standard. The full 12-item version was administered, consistent with psychometric evidence supporting the original subscale structure (Rice et al., 2019).

Perceived stress was measured using the Perceived Stress Scale (PSS-10; Cohen et al., 1983), whose Chinese version has shown good psychometric properties in Chinese samples (Lu et al., 2017). Psychometric studies have consistently distinguished a factor formed by the negatively worded items, which indexes perceived helplessness or distress, from a factor formed by the positively worded items, which indexes perceived self-efficacy or coping (Lee, 2012; Roberti et al., 2006). The present model concerns how negative states translate into a compensatory preference. This route is assumed to be triggered by distress rather than by appraised coping capacity. Only the six negatively worded items indexing perceived distress were, therefore, retained for analysis.

State anxiety was measured using the state anxiety subscale of the State-Trait Anxiety Inventory (STAI-S; Spielberger et al., 1983). The subscale contains 20 items and requires participants to report the degree of tension, worry, and uneasiness they feel “right now.”

Preference for ugly–cute memes was measured by participants’ rating of the item “How much do you like this type of image” after viewing the target ugly–cute meme image. Ratings of the control stimulus were collected to prevent participants from evaluating the ugly–cute image in isolation. They were not analyzed further.

Because all focal variables in Study 1 were self-reported, common method variance (CMV) was addressed primarily through procedural remedies (Podsakoff et al., 2003). Participants were assured of anonymity and told that there were no right or wrong answers; items indexing different constructs were interspersed and separated by task instructions; and the four measures used different response formats and anchors (seven-point agreement, five-point frequency, four-point intensity, and a five-point liking rating made after stimulus exposure), which reduce the scale-format component of method variance. Harman’s single-factor test is reported for comparability with prior work: the first unrotated factor accounted for 47.64% of the total variance, below the 50% criterion proposed by Podsakoff and Organ (1986).

### 2.5. Results

#### 2.5.1. Descriptive Statistics and Correlation Analysis

Pearson correlation analysis was used to examine relationships among variables. Means, standard deviations, and the correlation matrix are presented in Table 3. Gender was coded as 1 for male and 2 for female. Gender correlated positively with perfectionistic discrepancy, perceived stress, and state anxiety, indicating relatively higher scores among women. Regarding the core variables, perfectionistic discrepancy correlated positively with both perceived stress and state anxiety. Perceived stress also correlated positively with state anxiety (all *p*s < 0.01). However, the direct correlations of perfectionistic discrepancy, perceived stress, and state anxiety with ugly–cute meme preference did not reach significance.

#### 2.5.2. Mediation Effect Test

**Figure 2:**
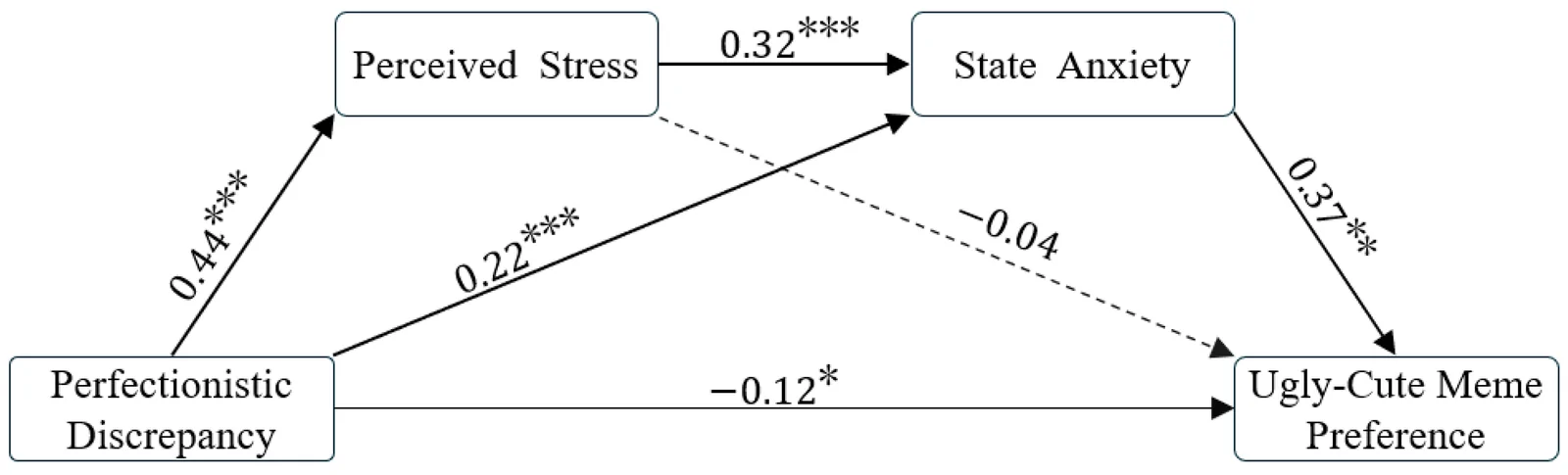
Serial mediation model of perfectionistic discrepancy in ugly–cute meme preference. * *p* < 0.05; ** *p* < 0.01; *** *p* < 0.001.

PROCESS Model 6 for SPSS 26.0 was used to test the serial mediation effects (Hayes, 2017; Igartua & Hayes, 2021). Variables entered the regression models on their original scales, and 95% percentile bootstrap confidence intervals for the indirect effects were estimated with 5000 resamples. Table 4 reports unstandardized regression coefficients (b); to facilitate comparison across indirect paths, Table 5 reports completely standardized indirect effects. The serial mediation model with path coefficients is presented in Figure 2. In Figure 2, solid arrows denote statistically significant paths, and dashed arrows denote nonsignificant ones.

The regression results are shown in Table 4. Perfectionistic discrepancy significantly and positively predicted perceived stress (*b* = 0.44, *p* < 0.001) and state anxiety (*b* = 0.22, *p* < 0.001), and perceived stress significantly and positively predicted state anxiety (*b* = 0.32, *p* < 0.001). When perceived stress and state anxiety were included simultaneously, the direct predictive effect of perfectionistic discrepancy on ugly–cute meme preference was significantly negative (*b* = −0.12, *p* < 0.05). State anxiety significantly and positively predicted ugly–cute meme preference (*b* = 0.37, *p* < 0.01), whereas perceived stress did not significantly predict ugly–cute meme preference (*b* = −0.04, *t* = −0.52). The total effect of perfectionistic discrepancy on ugly–cute meme preference was not significant (*c* = −0.01, *t* = −0.25, *p* = 0.80), whereas the direct effect after controlling for mediators was significantly negative (*c*′ = −0.12, *p* < 0.05). Thus, H1 was supported at the level of the direct effect. This pattern suggests that perfectionistic discrepancy is associated with ugly–cute meme preference through direct and indirect paths operating in opposite directions, indicating competitive mediation or a suppression effect (X. Zhao et al., 2010).

The mediation analysis is shown in Table 5. The separate indirect effect of perfectionistic discrepancy on ugly–cute meme preference through perceived stress was not significant (effect = −0.03, Boot *SE* = 0.06, 95% CI [−0.15, 0.08]), indicating that perceived stress alone did not function as a mediator; H2 was not supported. The indirect effect through state anxiety was significant (effect = 0.13, Boot *SE* = 0.04, 95% CI [0.05, 0.22]), supporting H3. The serial indirect effect through perceived stress and then state anxiety was also significant (effect = 0.09, Boot *SE* = 0.03, 95% CI [0.03, 0.14]), supporting H4.

In sum, Study 1 supported the hypothesized dual-path structure at the correlational level. Perfectionistic discrepancy was linked to ugly–cute meme preference negatively through the direct path and positively through anxiety-related indirect paths, with the two components canceling each other out in the total effect. Whether a stressful state genuinely pushes people toward ugly–cute content can only be settled by manipulating stress itself, which was the purpose of Study 2.

## 3. Study 2

### 3.1. Hypotheses

Study 2 used a randomized between-subjects design to test whether inducing stress raises preference for ugly–cute images. Stress was induced through autobiographical recall. In this task, participants retrieved and wrote about a personally experienced episode. The technique is widely used and validated, and it combines experimental control with personal relevance (Mills & D’Mello, 2014; Siedlecka & Denson, 2019). Accordingly, the following hypothesis was proposed:

**H5.**  *Compared with a neutral-recall condition, a stress-recall induction increases preference for ugly–cute images.*

If the induced preference shift reflects the emotional compensation mechanism, it should be associated with the state anxiety aroused by the stressor. This design tested a single proximal anxiety pathway linking the randomized stress induction to ugly–cute image preference. This reasoning yields a second hypothesis:

**H6.**  *State anxiety mediates the effect of the stress induction on ugly–cute image preference.*

### 3.2. Participants and Design

A total of 410 Chinese adults were recruited through the Wenjuanxing platform and completed the online experiment. Seventeen responses were excluded because they failed one or more quality criteria, including attention-check failure, abnormal completion time, noncompliant recall text, or missing key information. The final sample comprised 393 valid participants (valid response rate = 95.85%), including 158 men (40.20%) and 235 women (59.80%), with a mean age of 22.25 years (*SD* = 3.73). The platform randomly assigned participants to a stress-recall condition (*n* = 205) or a neutral-recall control condition (*n* = 188). The two conditions did not differ in gender composition, χ^2^(1) = 2.66, *p* = 0.103, or age, *t* = 0.87, *p* = 0.385. The procedure was covered by the same ethics approval as Study 1, and all participants provided informed consent before taking part.

### 3.3. Stimuli

Whereas Study 1 used memes that were actually circulating on Chinese social media, Study 2 required esthetic/ugly–cute pairs matched on everything except the manipulated feature, which naturally circulating memes cannot supply. The Study 2 stimuli are therefore referred to throughout as images: they operationalize the visual core of the ugly–cute construct—baby-schema cuteness cues combined with perceptual incongruity—without the captions, recognizable characters, and cultural references that memes carry. The candidate stimuli were generated in 2025 using the image-generation function of ChatGPT (GPT-4o, OpenAI) from text prompts alone. Within each pair, an esthetic and an ugly–cute version were generated from a shared prompt. The prompt specified a single centered animal illustration on a plain background, in a consistent rendering style and without text. The two prompts in a pair diverged only in how the target feature was described. The esthetic prompt called for well-proportioned and symmetrical features and a composed look. The ugly–cute prompt kept everything else the same, but asked for the target feature to appear mildly exaggerated, asymmetrical, or comically incongruous while staying endearing. Each ugly–cute version was then refined through additional text instructions until it conveyed the intended look, with all shared elements held constant.

**Figure 3:**
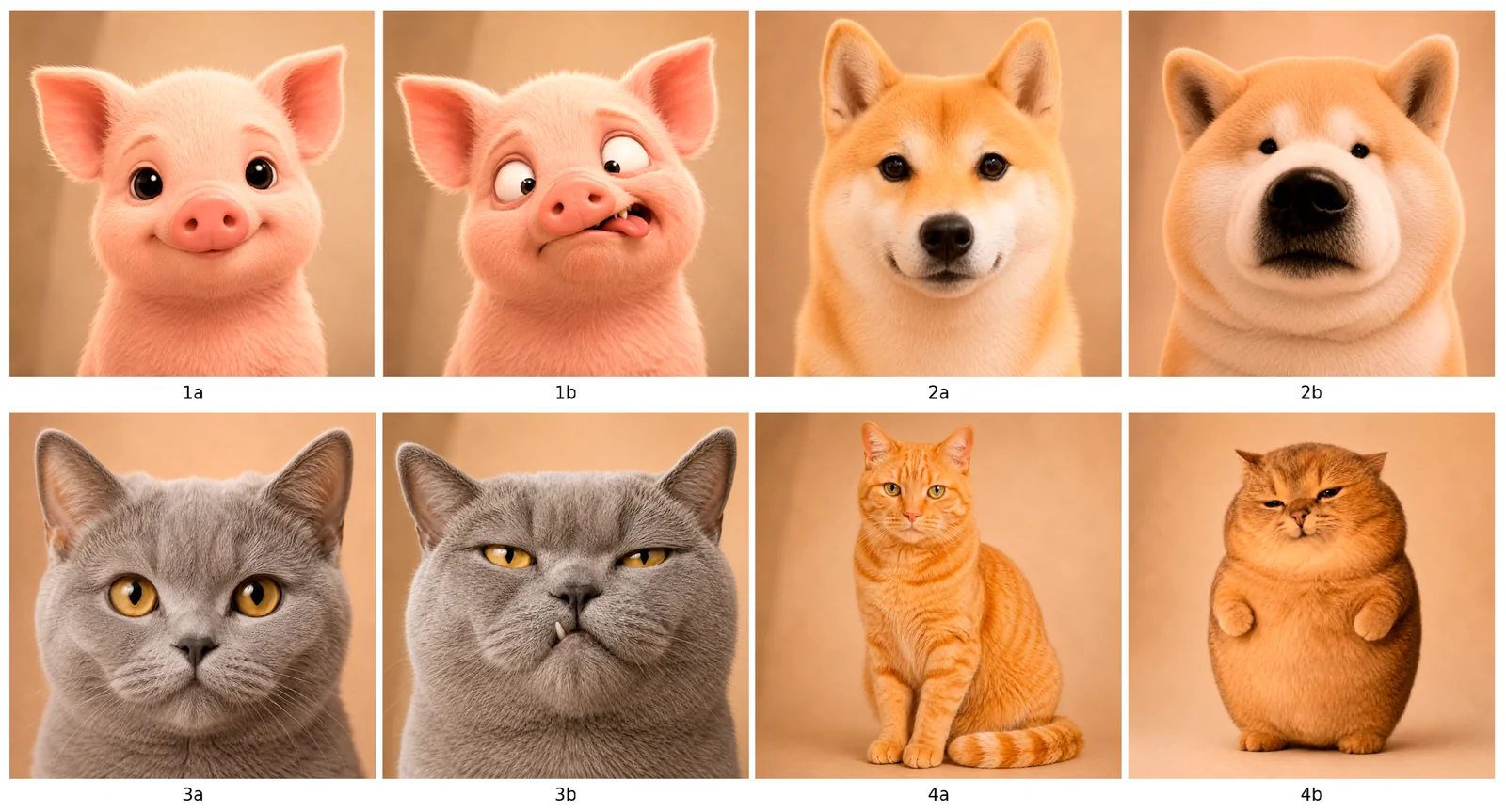
Candidate stimuli used in the Study 2 pretest (AI-generated). Each pair shows the esthetic version (**a**) and the ugly–cute version (**b**): (**1**) piglet, facial demeanor; (**2**) dog, facial features; (**3**) blue–gray cat, facial expression; and (**4**) ginger cat, body shape. The dog and ginger-cat pairs were retained for the formal experiment.

Stimuli for Study 2 were screened in a separate pretest. Four candidate esthetic/ugly–cute pairs were prepared, each manipulating ugly–cuteness through a different route. The four routes were facial demeanor (piglet), facial features (dog), facial expression (blue–gray cat), and body shape (ginger cat). Thirty-three participants drawn from the same population as the formal experiment rated every image on beauty, ugly–cuteness, familiarity, visual complexity, and willingness to save it as a sticker (1 = strongly disagree, 5 = strongly agree). Based on the screening results, the dog and ginger-cat pairs were retained as the critical stimuli for the formal experiment. Paired-sample *t*-tests confirmed that their esthetic versions were judged more beautiful than their ugly–cute versions (*M* = 4.15, *SD* = 0.74 vs. *M* = 2.83, *SD* = 1.10), *t*(32) = 5.40, *p* < 0.001, and lower in ugly–cuteness (*M* = 2.52, *SD* = 0.95 vs. *M* = 4.14, *SD* = 0.73), *t*(32) = −7.27, *p* < 0.001. The retained image types did not differ significantly in familiarity (*p* = 0.124), visual complexity (*p* = 0.108), or willingness to save them as stickers (*p* = 0.542). Figure 3 presents the four candidate pairs. Within a pair, the two versions share species, framing, background, and rendering style and differ only in the manipulated feature, meaning that any difference in evaluation can be attributed to that feature.

### 3.4. Procedure and Measures

Participants first rated their momentary stress and momentary anxiety on two 0–100 sliders (0 = not at all, 100 = extremely), and then completed the recall-writing task assigned to their condition (80–150 characters). Those in the stress-recall condition recalled a study or work task from the past 12 months completed under a pressing deadline and were asked to re-immerse themselves in the situation, whereas those in the control condition received parallel instructions to recall an ordinary recent activity completed at a normal pace. The two sliders were then readministered as a manipulation check.

Participants next completed a six-item, five-point state anxiety measure adapted from the State-Trait Anxiety Inventory (Spielberger et al., 1983; Marteau & Bekker, 1992), which served as the mediator (α = 0.91). In a subsequent simulated social-media browsing task, the four images from the two retained pairs were presented one per page in random order. Each image was rated on three five-point items (liking, willingness to like or collect the image, and willingness to save it as a sticker). These ratings were averaged into an ugly–cute image preference index (α = 0.89) and a parallel conventionally esthetic image preference index (α = 0.88). Participants then made two forced choices between the esthetic and the ugly–cute image of the same pair, rated the perceived mood-repair value of the images they had selected (1 = no effect at all, 5 = a very strong effect), and provided demographic information.

### 3.5. Results

#### 3.5.1. Manipulation Check

Table 6 summarizes the condition means. At baseline, the stress-recall and control conditions did not differ significantly in momentary stress (*M* = 39.36, *SD* = 23.64 vs. *M* = 35.32, *SD* = 24.99), *t* = 1.65, *p* = 0.101, *d* = 0.17, or momentary anxiety (*M* = 39.26, *SD* = 25.45 vs. *M* = 34.96, *SD* = 26.50), *t* = 1.64, *p* = 0.102, *d* = 0.17. After the recall task, the stress-recall condition reported higher momentary stress than the control condition (*M* = 54.14, *SD* = 23.98 vs. *M* = 30.43, *SD* = 24.73), *t* = 9.65, *p* < 0.001, *d* = 0.97, and higher momentary anxiety (*M* = 54.08, *SD* = 25.39 vs. *M* = 28.80, *SD* = 24.72), *t* = 9.99, *p* < 0.001, *d* = 1.01. The state anxiety scale showed the same pattern (*M* = 3.18, *SD* = 0.74 vs. *M* = 2.34, *SD* = 0.70), *t* = 11.55, *p* < 0.001, *d* = 1.17. These converging results indicate that the recall manipulation successfully increased stress-related states.

#### 3.5.2. Effects of the Stress Induction on Image Preference

To test H5, a 2 (condition: stress-recall vs. control) × 2 (image type: ugly–cute vs. conventionally esthetic) mixed-design analysis of variance was conducted. Neither the main effect of condition nor that of image type was significant, *ps* > 0.05. However, the interaction between condition and image type was significant, *F*(1, 391) = 27.62, *p* < 0.001. Follow-up simple-effects analyses showed that participants in the stress-recall condition rated the ugly–cute images more favorably than participants in the control condition, *p* < 0.001 (see Table 6). Preference for conventionally esthetic images was somewhat lower in the stress-recall condition, but the difference did not reach significance, *p* = 0.077.

The forced-choice task provided a secondary, choice-based index of preference. Across the two image pairs, participants were classified according to whether they chose the ugly–cute image on at least one of the two trials. A significantly higher proportion did so in the stress-recall condition than in the control condition (65.9% vs. 55.3%), χ^2^(1) = 4.14, *p* = 0.042. The number of ugly–cute choices correlated with the ugly–cute image preference index (*r* = 0.31, *p* < 0.001), providing evidence of convergence between the two preference measures.

As an exploratory follow-up, we examined how perceived mood-repair value related to image ratings within groups defined by forced-choice behavior. Among participants who selected the conventionally esthetic image in both trials, ratings of conventionally esthetic images were positively correlated with perceived mood-repair value, *r* = 0.39, *p* < 0.001, whereas among participants who selected the ugly–cute image in at least one trial, ratings of ugly–cute images were positively correlated with perceived mood-repair value, *r* = 0.31, *p* < 0.001. This pattern suggests that perceived mood-repair value tracks preference within both groups. It therefore does not, by itself, distinguish ugly–cute from conventional content as a regulation resource.

Pearson correlations are reported in Table 7. With gender coded as 1 = male and 2 = female, gender correlated negatively with ugly–cute image preference, indicating relatively lower preference scores among women. Ugly–cute image preference correlated positively with post-task momentary stress and state anxiety, and momentary stress correlated strongly with state anxiety.

#### 3.5.3. Mediation Analysis

**Figure 4:**
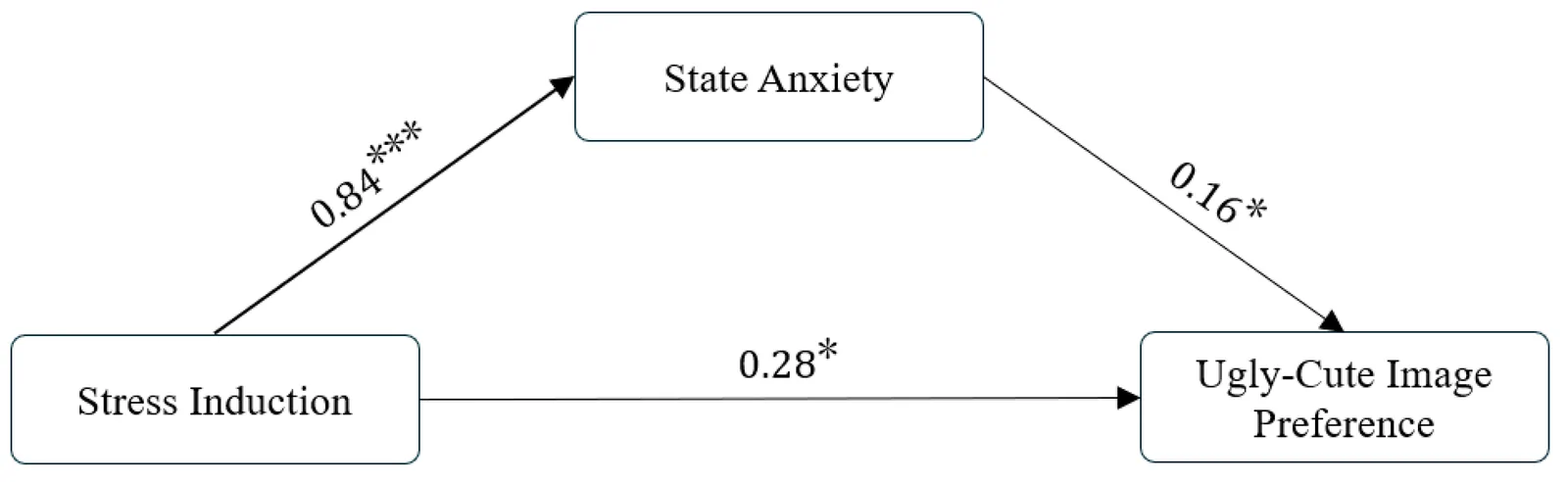
Mediation model of stress induction on ugly–cute image preference in Study 2. * *p* < 0.05; *** *p* < 0.001.

H6 was tested with experimental condition (1 = control, 2 = stress-recall) as the independent variable, state anxiety as the mediator, and the ugly–cute image preference index as the outcome. All coefficients are unstandardized, and 95% percentile bootstrap confidence intervals were computed from 5000 resamples. The regression results appear in Table 8, and the path model is shown in Figure 4. The stress-recall condition increased state anxiety (*a* = 0.84, *SE* = 0.07, *p* < 0.001), and its total effect on ugly–cute image preference was significant (*c* = 0.41, *SE* = 0.10, *p* < 0.001). With condition controlled, state anxiety positively predicted preference (*b* = 0.16, *SE* = 0.07, *p* = 0.018), and the direct effect of condition remained significant, although it was reduced (*c*′ = 0.28, *SE* = 0.11, *p* = 0.013). The indirect effect through state anxiety was significant (*ab* = 0.13, Boot *SE* = 0.06, 95% CI [0.02, 0.25]; Table 9). H6 was therefore supported. This pattern indicates complementary mediation, in which the direct and indirect effects point in the same direction (X. Zhao et al., 2010).

## 4. Discussion

### 4.1. Competing Pathways

The present research examined why ugly–cute memes appeal to some people more than others. It combined a cross-sectional study of perfectionistic discrepancy with a randomized stress induction. The findings of Study 1 are consistent with two opposing associations between perfectionistic discrepancy and ugly–cute meme preference, which we interpret as esthetic rejection and emotional compensation. Study 1 supported H1, H3, and H4, whereas H2 was not supported because perceived stress alone did not yield a significant indirect effect. Study 2 supported H5 and H6: stress recall increased ugly–cute image preference, and this effect was partially mediated by state anxiety. Because ugly–cute memes retain atypical, incongruous, or mildly defective features, they may lower initial processing fluency for individuals high in perfectionistic discrepancy. Such individuals are especially sensitive to these deviations and may therefore form stricter initial evaluations (Reber et al., 2004; Veryzer & Hutchinson, 1998).

According to models of esthetic processing, individuals typically pass through multiple stages, from perceptual analysis and classification to cognitive mastery and evaluation (Leder et al., 2004). Ugly–cute objects may also be re-evaluated after initial beauty–ugliness judgments through fun, uniqueness, and meaning interpretation (Li et al., 2024). The indirect associations through state anxiety and through the serial path were positive, whereas the direct association was negative; the total effect of perfectionistic discrepancy was close to zero. This is the pattern of competitive mediation, or suppression. A predictor lowers an outcome through one route while raising it through another, so that the zero-order relationship understates both processes. Such patterns have precedents in esthetic psychology. Typicality and novelty jointly predict esthetic preference for product designs, yet they mutually suppress each other. Neither correlates significantly with preference until the other is partialed out (Hekkert et al., 2003). The same pattern has been reported among the determinants of preference for product metaphors (Cila et al., 2014). Two implications follow. Theoretically, relying on the total effect alone would have suggested that perfectionistic discrepancy is unrelated to ugly–cute meme preference. In fact, it is related through two opposing routes. Mechanisms that cancel at the surface can be recovered only by modeling the direct and indirect paths jointly. Practically, the observed pattern identifies perfectionistic discrepancy and state anxiety as two measurable audience characteristics that can be included in future evaluations of responses to ugly–cute communication materials.

### 4.2. Experimental Evidence and Cross-Study Interpretation

In Study 1, the separate mediating effect of perceived stress was not significant, indicating that stress appraisal itself is insufficient to directly translate into stronger ugly–cute meme preference. In contrast, both the separate indirect effect of state anxiety and the serial indirect effect from perceived stress to state anxiety were significant. This result is consistent with the functional distinction between stress appraisal and anxiety response outlined above. Only the more proximal affective state translates into a compensatory preference. The positive indirect effect of state anxiety fits an emotion-regulation and compensatory account (Mandel et al., 2017), in which anxious individuals draw on the humor and low-threat cuteness of such content for mood repair.

Because the two studies used different stimulus types, the outcome is labeled ugly–cute meme preference in Study 1 and ugly–cute image preference in Study 2; both are treated as indicators of the same underlying construct, preference for ugly–cute content, with Study 1 contributing naturalistic relevance and Study 2 stimulus control. Study 2 extends the correlational findings with experimental evidence. Recalling a stressful deadline increased momentary stress, momentary anxiety, and state anxiety, and participants in the stress-recall condition rated ugly–cute images more favorably than did controls. By contrast, the reduction in preference for conventionally esthetic images did not reach significance. Accordingly, the experimental evidence points to a selective increase in the appeal of the ugly–cute images, reflected both in continuous ratings and in forced-choice preferences. Random assignment supports interpreting the rating difference as an effect of the recall manipulation, consistent with the emotional-compensation account that low-threat, humorous, and mildly absurd content may gain value under stress (Akram & Drabble, 2022; Mandel et al., 2017). The mediation results further support an anxiety-related mechanism. The induction raised state anxiety, which in turn predicted greater ugly–cute image preference.

Taken together, these findings contribute to research in esthetic psychology on the relationship between visual incongruity and esthetic preference. They suggest that the appeal of incongruent forms may depend not only on initial processing fluency but also on viewers’ transient affective states, possibly through emotion-dependent reappraisal following an initially disfluent response (Reber et al., 2004; Leder et al., 2004; Graf & Landwehr, 2017). From a consumer-psychology perspective, ugly–cute content may function as a low-threat symbolic resource whose value increases when self-discrepancy or anxiety creates a need for compensation (Graf & Landwehr, 2017; Mandel et al., 2017). From the perspectives of emotion regulation and digital communication, state anxiety appears to be a proximal condition in which humorous, cute, and mildly absurd memes acquire mood-repair value. The meaning and reception of meme-based messages may thus be co-produced by stimulus features and users’ transient affective states (Akram & Drabble, 2022; Shifman, 2013).

### 4.3. Limitations and Future Research

While the present research has produced some meaningful results, it is not without limitations.

First, a cross-sectional design was used in Study 1, and perfectionistic discrepancy was measured as an individual-difference variable. Therefore, its direct and indirect associations cannot establish temporal order or causal direction. The proposed esthetic-evaluation mechanism was also not directly tested, although perceived beauty and processing fluency are theoretically relevant to esthetic responses (Graf & Landwehr, 2017; Reber et al., 2004). In Study 2, random assignment supports a causal interpretation of the effect of the stress-recall condition on preference, but the measured mediator does not establish state anxiety as the causal mechanism (Bullock et al., 2010; Imai et al., 2010). Future research should combine longitudinal or cross-lagged designs with direct measures of esthetic evaluation and multi-method assessments of anxiety to clarify temporal ordering and the underlying mechanisms.

Second, the scope of the constructs restricts what can be concluded. Study 1 conceptualized and administered only the discrepancy facet; the design therefore cannot compare Discrepancy with High Standards, cannot test their interaction, and cannot address whether the observed pattern is specific to maladaptive perfectionists as classified by both facets (Rice & Ashby, 2007). Preference in Study 1 was also measured with a single item and a single stimulus, precluding an estimate of internal consistency and limiting generalization beyond that image. Future research should use multi-item preference measures and larger stimulus samples.

Third, the ecological validity of the stimuli and stress manipulation was limited. The AI-generated images used in Study 2 lacked the captions, recognizable characters, cultural references, and situational contexts of naturally circulating memes (Shifman, 2013). In addition, the recall task induced only mild and brief stress, and the lower ugly–cute preference reported by women in Study 2 was neither predicted nor replicated in Study 1. Future studies should test naturally circulating and dynamic forms of ugly–cute content under stressors of different intensities and durations, and examine the exploratory gender difference through preregistered replication.

Finally, both samples consisted of relatively young Chinese adults, and the Study 1 sample mainly comprised college students. The findings, therefore, may not generalize to other age groups, cultural backgrounds, or levels of engagement with online meme cultures. Meme interpretation depends partly on familiarity with shared cultural conventions, while emotion-regulation practices also vary across cultures (Matsumoto et al., 2008; Nissenbaum & Shifman, 2017). Cross-cultural and cross-generational replications using more demographically diverse, nonstudent samples are therefore needed.

## 5. Conclusions

Across a questionnaire study and a randomized experiment with Chinese adults, this research presents a two-sided account of the appeal of ugly–cute content. Study 1 suggests that perfectionistic discrepancy relates to ugly–cute meme preference through two opposing routes. The first is a negative direct association, consistent with a tendency to reject imperfect and atypical appearances. The second comprises positive indirect associations operating through anxiety. Study 2 shows that briefly recalling a stressful experience increased favorable ratings of ugly–cute images and raised the proportion of participants who chose an ugly–cute image when forced to choose, whereas the reduction in conventionally esthetic image ratings did not reach significance. The estimated indirect effect of the stress-recall condition on ugly–cute image preference through state anxiety was statistically significant. Together, these findings suggest that the appeal of ugly–cute content depends jointly on esthetic standards and on the affective state of the viewer.

## Figures and Tables

**Table 1:** Demographic characteristics (*n* = 356).

	Items	Number	Percentage (%)
Gender	Male	166	46.63
	Female	190	53.37
Age	18–20 years old	108	30.34
	21–23 years old	186	52.25
	24 years old and above	62	17.42
Usage Frequency	Occasionally	243	68.26
	Sometimes	102	28.65
	Frequently	11	3.09

**Table 2:** Questionnaires and sample items.

Variable	Scale Source	Items	Sample Item	α
Perfectionistic discrepancy (APS-R Discrepancy subscale)	APS-R (Slaney et al., 2001)	12	My best just never seems to be good enough for me.	0.91
Perceived stress (PSS-10 distress subscale)	PSS-10 (Cohen et al., 1983; Chinese version: Lu et al., 2017)	6	I felt difficulties were piling up so high that I could not overcome them.	0.89
State anxiety	STAI-S (Spielberger et al., 1983)	20	I feel nervous.	0.88
Ugly–cute meme preference	Self-developed single item	1	How much do you like this type of image?	-

Note: APS-R = Almost Perfect Scale–Revised; PSS-10 = Perceived Stress Scale-10; STAI-S = State-Trait Anxiety Inventory–State subscale; α = Cronbach’s alpha.

**Table 3:** Means, standard deviations, and correlations among variables (*n* = 356).

	*M*	*SD*	1	2	3	4	5
1. Gender	-	-	-				
2. Age	21.72	2.14	0.05	-			
3. Ugly–cute meme preference	3.66	0.98	0.05	0.03	-		
4. Perfectionistic discrepancy	3.76	1.60	0.17 **	0.08	−0.02	-	
5. Perceived stress	2.94	0.99	0.20 **	0.09	0.03	0.69 **	-
6. State anxiety	2.44	0.73	0.24 **	0.14 **	0.09	0.78 **	0.77 **

Note: ** *p* < 0.01.

**Table 4:** Mediation effects between perfectionistic discrepancy and ugly–cute meme preference.

	Perceived Stress			State Anxiety			Ugly–Cute Meme Preference		
	*b*	*SE*	*t*	*b*	*SE*	*t*	*b*	*SE*	*t*
Constant	1.30	0.09	13.45 ***	0.68	0.07	10.12 ***	3.33	0.18	18.16 ***
Perfectionistic discrepancy	0.44	0.02	18.46 ***	0.22	0.02	11.68 ***	−0.12	0.05	−2.33 *
Perceived stress				0.32	0.03	10.69 ***	−0.04	0.08	−0.52
State anxiety							0.37	0.13	2.92 **
*R* ^2^	0.49			0.70			0.03		
*F*	340.86			418.13			3.25		

Note: * *p* < 0.05; ** *p* < 0.01; *** *p* < 0.001.

**Table 5:** Completely standardized indirect effects with bootstrap 95% confidence intervals.

	Effect	Boot *SE*	Boot LLCI	Boot ULCI
Total Indirect Effect	0.19	0.07	0.05	0.33
Ind1: perfectionistic discrepancy → perceived stress → ugly–cute meme preference	−0.03	0.06	−0.15	0.08
Ind2: perfectionistic discrepancy → state anxiety → ugly–cute meme preference	0.13	0.04	0.05	0.22
Ind3: perfectionistic discrepancy → perceived stress → state anxiety → ugly–cute meme preference	0.09	0.03	0.03	0.14

Note: Values are completely standardized indirect effects; 5000 bootstrap resamples; percentile confidence intervals are reported.

**Table 6:** Condition means, standard deviations, and comparisons for all Study 2 measures (*n* = 393).

Measure	Control (*n* = 188)	Stress-Recall (*n* = 205)	*t*	*p*	*d*
Momentary stress (pre)	35.32 (24.99)	39.36 (23.64)	1.65	0.101	0.17
Momentary stress (post)	30.43 (24.73)	54.14 (23.98)	9.65	<0.001	0.97
Momentary anxiety (pre)	34.96 (26.50)	39.26 (25.45)	1.64	0.102	0.17
Momentary anxiety (post)	28.80 (24.72)	54.08 (25.39)	9.99	<0.001	1.01
State anxiety	2.34 (0.70)	3.18 (0.74)	11.55	<0.001	1.17
Ugly–cute image preference	3.28 (1.03)	3.69 (0.86)	4.26	<0.001	0.43
Conventionally esthetic image preference	3.50 (0.96)	3.34 (0.84)	−1.77	0.077	−0.18

Note: Values are *M* (*SD*). Comparisons are Welch’s *t*-tests; momentary ratings were given on 0–100 sliders.

**Table 7:** Means, standard deviations, and correlations among variables in Study 2 (*n* = 393).

	*M*	*SD*	1	2	3	4	5
1. Gender	–	–	–				
2. Age	22.25	3.73	−0.06	–			
3. Ugly–cute image preference	3.50	0.97	−0.17 **	0.01	–		
4. Momentary stress (post-task)	42.80	27.05	0.03	0.12 *	0.20 **	–	
5. State anxiety	2.78	0.84	0.03	0.08	0.21 **	0.74 **	–

Note: * *p* < 0.05; ** *p* < 0.01.

**Table 8:** Regression models for experimental mediation in Study 2.

	State Anxiety			Ugly–Cute Image Preference		
	*b*	*SE*	*t*	*b*	*SE*	*t*
Constant	1.50	0.11	12.81 ***	2.64	0.18	14.60 ***
Condition	0.84	0.07	11.55 ***	0.28	0.11	2.50 *
State anxiety				0.16	0.07	2.37 *
*R* ^2^	0.25			0.06		
*F*	133.39			11.97		

Note: Condition was coded as 1 = control and 2 = stress-recall. The mediator was the state anxiety scale. * *p* < 0.05; *** *p* < 0.001.

**Table 9:** Total, direct, and indirect effects for the experimental mediation in Study 2.

Path	Effect	Boot *SE*	Boot LLCI	Boot ULCI
Total effect: condition → ugly–cute image preference (*c* path)	0.41	0.10	0.22	0.59
Direct effect: condition → ugly–cute image preference, state anxiety controlled (*c*′ path)	0.28	0.11	0.06	0.49
Indirect effect: condition → state anxiety → ugly–cute image preference (*ab* path)	0.13	0.06	0.02	0.25

Note: 5000 bootstrap resamples; percentile confidence intervals are reported.

## Data Availability

The data presented in this study are available from the corresponding authors upon reasonable request due to privacy.
